# Reliability of comprehensive echocardiography evaluation of patent ductus arteriosus among extremely preterm neonates across a national network: A prospective observational study

**DOI:** 10.1177/19345798251349744

**Published:** 2025-07-24

**Authors:** Dany E Weisz, Laura Thomas, Xiang Y Ye, Luc Mertens, Anie Lapointe, Andréanne Villeneuve, Gabriel Altit, Renjini Lalitha, Nadya Ben Fadel, George Jacob, Deepak Louis, Soume Bhattacharya, Amuchou Soraisham, Audrey Hébert, Souvik Mitra, Abbas Hyderi, Joseph Y Ting, Michael Castaldo, Aimann Surak, Poorva Deshpande, Yasser Elsayed, Bonny Jasani, Sagee Nissimov, Faith Zhu, Prakesh Shah, Amish Jain

**Affiliations:** 1 Newborn and Developmental Paediatrics, 71545 Sunnybrook Health Sciences Centre, Toronto, Ontario, Canada; 2 Department of Paediatrics, University of Toronto, Toronto, Ontario, Canada; 3 Department of Paediatrics, 518775 Mount Sinai Hospital, Toronto, Ontario, Canada; 4 Maternal-Infant Care Research Centre, 518775 Mount Sinai Hospital, Toronto, Ontario, Canada; 5 Division of Paediatric Cardiology, 27338 Hospital for Sick Children, Toronto, Ontario, Canada; 6 Division of Neonatology, CHU Sainte Justine, Montréal, Quebec, Canada; 7 Division of Neonatology, Montreal Children’s Hospital, Montreal, Quebec, Canada; 8 Division of Neonatology, London Health Sciences Centre, London, Ontario, Canada; 9 Division of Neonatology, Children’s Hospital of Eastern Ontario, Ottawa, Ontario, Canada; 10 Division of Neonatology, St. Boniface Hospital, Winnipeg, Manitoba, Canada; 11 Division of Neonatology, Alberta Children’s Hospital, Calgary, Alberta, Canada; 12 Division of Neonatology, CHU du Quebec, Quebec, Canada; 13 Division of Neonatology, IWK Hospital, Halifax, Nova Scotia, Canada; 14 Division of Neonatal Care, Department of Pediatrics, 3158 University of Alberta, Edmonton, Alberta, Canada; 15 Division of Neonatology, British Columbia Children’s Hospital, Vancouver, British Columbia, Canada; 16 Division of Neonatology, Hospital for Sick Children, Toronto, Ontario, Canada

**Keywords:** coefficient of variation, intraclass correlation coefficient, kappa statistic, patent ductus arteriosus, PDA, premature, targeted neonatal echocardiography, TNE

## Abstract

**Background:**

Prior studies on the reliability of targeted neonatal echocardiography (TNE) among extremely low gestational age neonates (ELGANs) with a patent ductus arteriosus (PDA) have been limited to evaluation of pre-defined images by a small set of study observers. The objective of this study was to investigate the interobserver reliability of comprehensive echocardiography measures of PDA size/shunt volume and ventricular performance among TNE-neonatologists in a large national network.

**Methods:**

We conducted a prospective observational study. TNEs performed for the evaluation of PDA among ELGANs were analyzed by TNE-neonatologists from the Canadian TNE Consortium. Analyses were conducted via an interactive videoconferencing platform offering full remote control to the review software. Reliability for continuous measures was evaluated using the intraclass correlation coefficient (ICC) and coefficient of variation. The kappa statistic was used to evaluate the interobserver reliability of categorical parameters.

**Results:**

Reliability was excellent among indices of PDA size and gradient (ICC≥0.91) and good-to-excellent among most indices of left ventricular (LV) size and output (ICC≥0.79, except for LV end-systolic volume and left atrium to aortic root ratio). There was substantial to near-complete agreement on PDA shunt direction and diastolic flow abnormalities in the abdominal aorta and systemic arteries (kappa ≥0.78). However, reliability for measures of LV systolic and diastolic performance was variable with ICC range 0.21–0.95, though with low coefficient of variation (<15%).

**Conclusions:**

Interobserver reliability for most TNE measures of PDA size, gradient, shunt volume, and LV dimensions and function is good-to-excellent, supporting the validity of incorporating these indices in prospective multicenter research.

## Introduction

Patent ductus arteriosus (PDA) occurs in over 60% of extremely low gestational age (GA) neonates (ELGANs, defined as GA ≤ 27^+6^ weeks) and is associated with increased mortality and morbidity.^1^ However, there remains significant uncertainty regarding which neonates with persistent PDA may benefit from treatment. The American Academy of Pediatrics has called for the development of comprehensive echocardiography-based PDA risk stratification tools,^2^ whose development requires large, multicenter studies, ideally with prospectively collected echocardiography data. The RESET-PDA study (REdefining the SignificancE and Treatment threshold for PDA in ELGANs) is a prospective observational study being conducted among 17 tertiary neonatal intensive care units (NICUs) with established targeted neonatal echocardiography (TNE) programs across Canada through collaboration with the Canadian Neonatal Network. The study aims to develop postnatal-age specific definitions of hemodynamically significant PDA for ELGANs by incorporating comprehensive clinical and echocardiography data, to define potential treatment thresholds which identify ELGANs with PDA at higher risk of adverse neonatal outcomes.

While the abstraction of clinical data in the Canadian Neonatal Network has been shown to be highly reliable,^3^ the reliability of collecting a large set of functional echocardiography measurements from many operators across centers is not known. Prior studies investigating the reliability of echocardiography evaluation of neonates have been limited to evaluation of pre-specified images by a small set of study observers and/or lack of inclusion of extremely preterm neonates with PDA.^4–10^ This study was conducted as part of the RESET-PDA program, to investigate the interobserver reliability of measuring TNE variables representing PDA size and shunt volume, and left and right ventricular dimensions and function among expert-neonatologists across a national network.

## Methods

We conducted a prospective observational study including neonatologists within the Canadian TNE Consortium, a national group of neonatologists with established expertise and active practice in TNE programs in tertiary NICUs. For this study, consecutive TNEs performed at the NICU of Sunnybrook Health Sciences Centre using a standardized imaging protocol based on the American Society of Echocardiography’s guidelines for TNE^11^ were anonymized and selected for review. All included TNEs were performed for the assessment of PDA among ELGANs, using a Vivid E9 ultrasound machine and a 12 MHz phased array probe (General Electric™, Boston, USA). Consecutively performed TNEs during a randomly selected 1 month period (July 1 to July 31, 2022) were used to reflect pragmatic clinical practice with respect to echocardiographer, image quality, and image sequences. Among neonates with multiple echocardiograms, only the first was used to avoid dependency of the echocardiography measurements. Prior to undertaking this study and as part of the RESET-PDA program, the Canadian TNE Consortium held 3 virtual meetings to develop a consensus on measurement techniques for TNE variables, which conformed to published international guidelines.^12^

### Echocardiography analysis

Review of images was conducted via a secure, interactive, online videoconferencing platform (Zoom Video Communications™, San Jose, USA). To facilitate the evaluation of echocardiography measurement reliability under pragmatic, real-world conditions, the participants were provided the complete scan and full remote control of the echocardiography workstation and software (EchoPAC Suite, Viewpoint 6, GE, Boston, USA). For each TNE, participants were asked to perform analyses to estimate measures of PDA size and Doppler-derived velocity gradient, left ventricular (LV) dimensions and systolic and diastolic performance, right ventricular (RV) dimensions and systolic performance, and RV-pulmonary artery coupling.^13^ Pulse- (PW) and continuous-wave (CW) Doppler and M-mode measurements were performed in triplicate (3 consecutive cardiac cycles) and averaged, while B-mode measurements were performed once only (Table 1). All analyses were performed manually without the use of software semi-automation. As part of the pragmatic study design, participants were allowed to review the whole scan, select the images they deemed most appropriate to analyze, and omit measurements that they deemed non-feasible due to suboptimal image quality. To ensure blinding of the study data, all measurements were deleted from the software worksheet after each analysis.

**Table 1.** Technique and description of the performance of echocardiography measurements.

Echocardiography Variable(s)	Technique and echo view	Formula / description
Patent Ductus Arteriosus		
PDA diameter	2D and color Doppler (simultaneous) “Ductal cut” (modified high parasternal short axis view) demonstrating the entire length of the PDA from aorta to pulmonary artery	Ductal diameter estimated as the narrowest diameter on 2D imaging
PDA gradient	Ductal cut view. PW-Doppler (if peak velocity < 2 m/s) with sample volume exclusively in PDA at pulmonary end; CW-Doppler (if peak velocity > 2 m/s) with Doppler beam through the entire length of the PDA.	PDA gradient traced from onset of ventricular systole (QRS complex on ECG). Peak systolic gradient estimated as the largest Ao-PA pressure difference in systole. Mean gradient estimated as the mean Ao-PA pressure difference over the full cardiac cycle (systole and diastole). End-diastolic velocity is the lowest velocity at end diastole, estimated using point caliper
Peak systolic gradient		
Mean gradient		
End diastolic velocity		
PDA shunt direction	PW or CW Doppler of transductal gradient	Categorical classification:
		(1) Exclusively left-to-right shunt
		(2) Mostly left-to-right shunt (defined as pulmonary-to-systemic shunting comprising <30% of the cardiac cycle by time)
		(3) Bidirectional shunt (defined as pulmonary-to-systemic shunting comprising ≥30% of the cardiac cycle by time)
Left Ventricle Dimensions and Output		
LVEDV	2D	LVEDV and LVESV: Biplane estimated as the mean of the 4-chamber and 2-chamber LVEDV and LVESV, respectively
LVESV	Apical 4 and 2 chamber views	LVEF (biplane) estimated as (LVEDV – LVESV) / LVEDV
LV EF (Simpson’s biplane) (%)	Manual tracing of LV endocardial border–blood interface at end diastole and end systole	
LVIDd	M-mode	LVIDd: Internal dimension of LV at end diastole
LVIDs	PSAX	LVIDs: Internal dimension of LV at end systole
LV SF (%)		LV SF (%) = (LVEDd – LVEDs) / LVEDd * 100
LVOT diameter	2D	Distance between aortic valve hinge-points at peak systole
	PLAX	
LVOT VTI	PW Doppler	Sample volume placed at the level of the aortic valve
	Apical 5-chamber view	
Left Ventricle Systolic and Diastolic Performance		
Mitral valve inflow velocities: E, a (cm/s)	PW Doppler	Sample volume placed immediately distal to the tips of the valve leaflets
	Apical 4-chamber	
LV IVRT (msec)	PW Doppler	Sample volume placed midway between the AoV and MV to obtain a clear signal showing both aortic outflow and mitral inflow
	Apical 3-chamber	
LV and septal TDI peak velocities: e’, a’, s’ (cm/s)	TDI	1–2 mm sample volume on the basal segment of the lateral and septal walls just below the mitral valve annulus
	Apical 4-chamber	
Right Ventricle Dimensions, Systolic Performance, and Ventricular-Arterial Coupling		
RVEDA	2D	RVEDA and RVESA obtained by tracing RV endocardial borders at end-diastole and end-systole, respectively
RVESA	RV focused apical four-chamber	RV FAC (%) = (EDA – ESA) / EDA x 100
RV FAC (%)		
TAPSE (mm)	M-mode	Line of interrogation passing through the lateral aspect of the TV annulus while maintaining vertical alignment with the apex
	RV focused apical four-chamber	
PAAT	PW Doppler	Sample volume placed distal to pulmonary valve in MPA
RVET	PSAX	From Doppler envelope, using time caliper, estimate time from initiation of RV ejection to peak velocity (PAAT) and to termination of RV ejection (RVET)
Diastolic Flow in Systemic Arteries		
Abdominal aorta holodiastolic flow reversal	PW Doppler of abdominal aorta at the level of the diaphragm	Categorical classification:
		(1) Present: Holodiastolic flow reversal considered present if reverse flow above the baseline seen throughout diastole
	Suprasternal/Arch view	(2) Not present: Holodiastolic flow reversal considered to not be present if reverse flow above the baseline was not seen throughout diastole
Celiac artery diastolic flow	PW Doppler of celiac artery with 1–2 mm sample volume	Categorical classification (1) antegrade: Antegrade diastolic flow throughout diastole in ≥50% of cardiac cycles
	Subcostal long axis view	(2) Reverse: Any flow reversal in diastole noted in ≥50% of cardiac cycles
	Minimum 7 cardiac cycles	(3) Absent: Absent diastolic flow in ≥50% of cardiac cycles, or if does not meet criteria for either antegrade or reverse categories
Middle cerebral artery diastolic flow	PW Doppler of the middle cerebral artery	Categorical classification:
		(1) Antegrade: Antegrade diastolic flow throughout diastole in ≥50% of cardiac cycles
	Sphenoid fontanelle	(2) Reverse: Any flow reversal in diastole noted in ≥50% of cardiac cycles
	Minimum 7 cardiac cycles	(3) Absent: Absent diastolic flow in ≥50% of cardiac cycles, or if does not meet criteria for either antegrade or reverse categories

*Note:* Sample volume for PW Doppler measurements set at 2 mm unless otherwise specified.

Abbreviations: A, late diastolic velocity (with atrial contraction); Ao, aorta; CW, continuous-wave; E, early diastolic velocity; IVRT, isovolumic relaxation time; LV, left ventricle; LV EF, left ventricle ejection fraction; LVEDV, left ventricle end-diastolic volume; LVESV, left ventricle end-systolic area; LVIDd, left ventricle internal diameter in diastole; LVIDs, left ventricle internal diameter in systole; LVOT, left ventricular outflow tract; LV SF, left ventricle shortening fraction; RV, right ventricle; RVEDA, right ventricular end-diastolic area; RVESA, right ventricular end-systolic area; RVFAC, right ventricle fractional area change; PA, pulmonary artery; PAAT, pulmonary artery acceleration time; PDA, patent ductus arteriosus; PW, pulse-wave; RVET, right ventricular ejection time; TAPSE, tricuspid annular plane systolic excursion; TDI, tissue-Doppler imaging; TV, tricuspid valve; VTI, velocity-time integral.

### Sample size and statistical analysis

Sample size determination was based on requirements for detecting the intraclass correlation coefficient (ICC) ≥ 0.80 for continuous TNE parameters, representing excellent reproducibility in neonates.^14^ To estimate an ICC of 0.80 with 95% confidence interval (CI) ± 0.15,^15^ a sample of at least 12 echocardiograms was required with 7 observers per echocardiogram. To achieve adequate power for this study, each of the 12 TNEs was analyzed by 7 different observers, randomly chosen from the pool of study participants using a computerized matching algorithm without resampling. Inclusion of a randomly selected subset of observers^8,16^ allowed for pragmatic evaluation and generalizability of study findings,^17^ while improving study feasibility by reducing participant burden.^18^

Descriptive characteristics were presented as mean (standard deviation) or median [interquartile range] for parametric and non-parametric continuous variables, respectively. Categorical data was presented as frequency and percentage. The ICC and the unweighted kappa statistic (κ) for multiple (>2) raters were used to evaluate the interobserver reliability of continuous and categorical parameters, respectively. A one-way random effects model was used to estimate the ICC.^19^ The ICC for reliability was interpreted as poor (ICC <0.4), moderate (0.4 ≤ ICC <0.6), good (0.6 ≤ ICC <0.8), and excellent (ICC ≥0.80).^8^ The kappa statistic (κ) for agreement of categorical measures was interpreted as poor (κ < 0.4), moderate (0.4 ≤ κ < 0.6), substantial (0.6 ≤ κ < 0.8), and near-complete (κ ≥ 0.80).^20^ The with-in subject coefficient of variation (CV) was estimated for continuous parameters, by computing the CV for each infant and then averaging across all infants. A CV < 15% was interpreted as good reproducibility.^21^ Analyses were conducted using SAS version 9.4 (Cary, USA). SAS macros %intracc with bootstrap method (100 iterations) and %magree were used for the estimation of ICC coefficients (95% CI) and κ, respectively. For each TNE variable, we evaluated the potential influence of perceived feasibility of acquisition of measurements by operators on reliability, by estimating the linear correlation of the ICC with the number of measurements omitted by the raters per echocardiography parameter. This study was approved by the local hospital Research Ethics Board (Project ID 5104) and was performed in accordance with the Declaration of Helsinki.

## Results

### Demographics

Among the 24 Canadian TNE neonatologists approached for the study, 21 (88%) agreed to participate. Of the participating neonatologists, the mean (±SD) age was 41 (±4) years, all had completed formal training programs in neonatal hemodynamics and targeted neonatal echocardiography (NHTNE), and the time since completion of NHTNE training was 5 (±3) years. Each TNE-neonatologist was randomly assigned to analyze 4 of the 12 TNEs, for a total of 84 evaluations (7 raters per TNE). All participants were active TNE practitioners representing 16 out of 17 tertiary NICUs in the Canadian TNE Consortium. The majority (69%) of NICUs were characterized as having both inborn and outborn neonates and neonates requiring surgical treatment.

The 12 consecutive TNEs included were performed among 12 different ELGANs by 3 different operators (two neonatologists and one trained sonographer, each of whom performed 4 of the TNEs). These neonates were born at GA 26.4 [24.7, 27.0] weeks, and had a weight and postnatal age at TNE of 856 [793, 941] grams and 18^10,21^ days, respectively. Half of the TNEs were performed with the patient receiving mechanical ventilation (all high frequency ventilation), while the remainder were performed while receiving non-invasive positive pressure support.

### Measurement reliability

**Figure 1.**
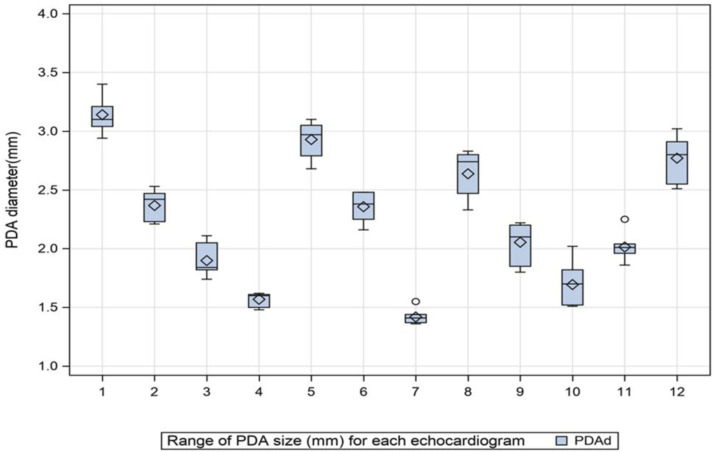
Graphical plot of the set of the estimates of PDA diameter for each of the 12 echocardiograms analyzed in the study, demonstrating the range of PDA sizes and variability in estimates.

Overall, TNE indices of PDA size and transductal gradient demonstrated excellent reliability (all ICC >0.9 and CV < 16%) (Table 2). Patent ductus arteriosus diameter among the 12 echocardiograms, as estimated by the analyzing neonatologists, ranged from 1.42 (±0.06) mm to 3.14 (±0.15) mm (Figure 1). Most indices of LV and RV dimensions and output also had good to excellent reliability, with ICC range 0.64–0.93, though CV was more variable, ranging from 3.6% to 31.8% (Table 2). The CV was >15% for LV/RV volumes/areas but <15% for the remaining parameters of chamber size. The reliability of echocardiography indices of LV and RV systolic and diastolic performance was highly variable (ICC range 0.21–0.95), with most parameters, except for mitral inflow and LV output, having poor to good reliability based on the ICC (Table 3). On examining within subject variability using the CV, however, most measures demonstrated good reproducibility, with CV < 15%.

**Table 2.** Interobserver reliability of echocardiography parameters of patent ductus arteriosus size and gradient, ventricular dimensions, and left ventricular output.

Echocardiography parameter	No. of subjects with measurements / no. of measurements performed (N/n)	Mean (SD)	Within subject coefficient of variation (%)	Intraclass correlation coefficient (95% CI)
Patent ductus arteriosus				
PDA diameter (mm)	12 / 84	2.24 (0.55)	6.3%	0.91 (0.86, 0.95)
PDA peak systolic gradient (mmHg)	11^a^ / 77	16.2 (8.8)	11.8%	0.95 (0.80, 0.98)
PDA mean gradient (mmHg)	11^a^ / 77	8.5 (6.7)	14.0%	0.97 (0.86, 0.99)
PDA minimum diastolic velocity (m/s)	11^a^ / 77	0.78 (0.54)	15.8%	0.91 (0.80, 0.96)
Left ventricle: Dimensions and velocity-time integral				
LV end diastolic volume (4 chamber) (mL)	12 / 84	2.75 (1.25)	17.6%	0.80 (0.69, 0.89)
LV end systolic volume (4 chamber) (mL)	12 / 84	1.12 (0.59)	29.7%	0.80 (0.69, 0.89)
LV end diastolic volume (2 chamber) (mL)	12 / 84	3.65 (2.12)	21.1%	0.79 (0.70, 0.83)
LV end systolic volume (2 chamber) (mL)	12 / 84	1.35 (0.79)	31.8%	0.69 (0.46, 0.75)
LV end diastolic volume (Biplane) (mL)	12 / 84	3.25 (1.63)	17.4%	0.82 (0.75, 0.85)
LV end systolic volume (Biplane) (mL)	12 / 84	1.35 (0.76)	25.4%	0.74 (0.56, 0.79)
LV internal diameter, diastole (PSAX) (mm)	12 / 84	14.4 (2.1)	3.7%	0.93 (0.80, 0.97)
LV internal diameter, systole (PSAX) (mm)	12 / 84	9.5 (1.5)	5.5%	0.86 (0.65, 0.91)
Left atrium to aortic root ratio	12 / 84	1.65 (0.33)	11.8%	0.65 (0.23, 0.79)
LV outflow tract velocity time integral (cm)	12 / 84	12.1 (3.7)	10.3%	0.83 (0.33, 0.96)
Heart rate (bpm)	12 / 84	165 (12)	2.6%	0.84 (0.55, 0.95)
LV outflow tract diameter (mm)	12 / 84	4.82 (0.72)	7.1%	0.77 (0.46, 0.86)
Right ventricle dimensions				
RV end diastolic area (4-chamber) (cm^2^)	12 / 84	1.54 (0.64)	16.9%	0.80 (0.62, 0.87)
RV end systolic area (4-chamber) (cm^2^)	12 / 84	0.97 (0.43)	20.4%	0.72 (0.49, 0.82)
RV internal diameter, diastole (PSAX) (mm)	12 / 84	5.0 (1.4)	14.1%	0.64 (0.27, 0.86)

a One patient had a bidirectional PDA shunt for whom PDA gradient was not estimated.

bpm, beats per minute; cm, centimeter; LV, left ventricle; mm, millimeter; PSAX, parasternal short axis; RV, right ventricle.

**Table 3.** Interobserver reliability of indices of left and right ventricular function and calculated parameters.

Echocardiography parameter	No. of subjects with measurements / no. of measurements performed (N/n)	Mean (SD)	Within subject coefficient of variation (%)	Intraclass correlation coefficient (95% CI)
Left ventricle: Systolic and diastolic performance				
Mitral valve E (cm/s)	12 / 81	65 (18)	4.9%	0.92 (0.43, 0.98)
Mitral valve A (cm/s)	12 / 81	76 (20)	6.7%	0.95 (0.53, 0.98)
LV septal e’ (m/s)	12 / 71	4.7 (1.0)	10.9%	0.21 (−0.06, 0.64)
LV septal a’ (m/s)	12 / 71	6.1 (1.2)	6.8%	0.66 (0.45, 0.80)
LV septal s’ (m/s)	12 / 71	4.5 (0.7)	5.3%	0.70 (0.45, 0.82)
LV lateral e’ (m/s)	12 / 80	5.4 (1.3)	13.4%	0.45 (0.25, 0.69)
LV lateral a’ (m/s)	12 / 80	6.2 (1.2)	13.1%	0.54 (0.26, 0.85)
LV lateral s’ (m/s)	12 / 84	4.6 (0.7)	6.5%	0.77 (0.63, 0.89)
Isovolumic relaxation time (msec)	12 / 81	38 (10)	14.2%	0.68 (0.32, 0.76)
Pulmonary vein S (m/s)	12 / 74	0.38 (0.11)	7.4%	0.30 (0.03, 0.76)
Pulmonary vein D (m/s)	12 / 74	0.40 (0.13)	11.8%	0.41 (0.03, 0.68)
Right ventricle systolic performance and ventricular-arterial coupling				
TAPSE (mm)	12 / 84	6.90 (1.95)	9.9%	0.75 (0.47, 0.86)
PAAT (msec)	12 / 84	44 (13)	20.6%	0.38 (0.03, 0.67)
RV ejection time (msec)	12 / 82	172 (28)	12.5%	0.23 (0.03, 0.50)
Calculated Parameters				
PDA: ratio of peak systolic velocity : Minimum diastolic velocity	11^a^ / 77	3.50 (2.24)	23.7%	0.46 (0.22, 0.93)
Mitral valve E:A ratio	12 / 81	0.87 (0.19)	4.4%	0.95 (0.36, 0.98)
Ejection fraction (biplane, %)	12 / 84	59.6 (6.0)	8.2%	0.24 (0.12, 0.35)
Fractional shortening (%)	12 / 84	33.9 (6.9)	12.2%	0.61 (0.36, 0.77)
LV septal E/e’	12 / 68	14.3 (5.3)	9.4%	0.56 (0.24, 0.84)
LV lateral E/e’	12 / 78	13.2 (5.4)	18.0%	0.42 (0.12, 0.66)
Left ventricular output (ml/min)	12 / 84	121 (61)	19.3%	0.84 (0.50, 0.90)
Pulmonary vein S:D ratio	12 / 74	1.07 (0.45)	14.2%	0.27 (0.002, 0.73)
Ratio of PAAT : RV ejection time	12 / 82	4.2 (1.3)	19.7%	0.45 (0.26, 0.63)

^a^One patient had a bidirectional PDA shunt for whom PDA gradient was not estimated.

A, late diastole; D, diastole; E, early diastole; LV, left ventricle; PAAT, pulmonary artery acceleration time; PDA, patent ductus arteriosus; RV, right ventricle; S, systole; TAPSE, tricuspid annular plane systolic excursion.

There was substantial to near-complete agreement on PDA shunt direction and diastolic flow abnormalities in the abdominal aorta, celiac artery, and middle cerebral artery (Table 4). For celiac artery diastolic flow, ICC was 0.78 (95% CI: 0.69, 0.87) for the ternary classification (antegrade /absent /reverse flow) but improved to 1.00 (95% CI: 0.87, 1.00) by adopting a binary classification of normal (antegrade) or abnormal (absent or reverse). For middle cerebral artery flow, the ICC for both ternary and binary classifications demonstrated complete agreement (ICC 1.00, 95% CI: 0.87, 1.00).

**Table 4.** Agreement of nominal ratings of echocardiography parameters of patent ductus arteriosus shunt direction and diastolic flow abnormalities of the abdominal aorta, celiac artery, and middle cerebral artery.

Echocardiography parameter	Classification	Category-specific kappa statistic (95% CI)	Overall kappa statistic (95% CI)
PDA shunt direction	Exclusively left to right	0.79 (0.67, 0.91)	0.79 (0.69, 0.89)
	Mostly left to right	0.47 (0.35, 0.59)	
	Bidirectional	1 (0.87, 1)	
Abdominal aorta diastolic flow reversal	Holodiastolic flow reversal	—	0.80 (0.68, 0.92)
	No holodiastolic flow reversal		
Celiac artery diastolic flow^a^	Reverse diastolic flow	0.61 (0.48, 0.74)	0.78 (0.69, 0.87)
	Absent antegrade flow	0.68 (0.55, 0.81)	
	Antegrade flow	1 (0.87, 1)	
Middle cerebral artery diastolic flow^b^	Reverse diastolic flow	1 (0.87, 1)	1 (0.90, 1)
	Absent antegrade flow	1 (0.87, 1)	
	Antegrade flow	1 (0.87, 1)	

^a^Celiac artery diastolic flow: ICC 1 (0.87, 1) when considered as a binary category (“Normal” (antegrade diastolic flow) versus “Abnormal” (absent or reverse diastolic flow)).

^b^Middle cerebral artery diastolic flow: ICC 1 (0.87, 1) when considered as a binary category (“Normal” (antegrade diastolic flow) versus “Abnormal” (absent or reverse diastolic flow)).

### Quality of echocardiography images

**Figure 2.**
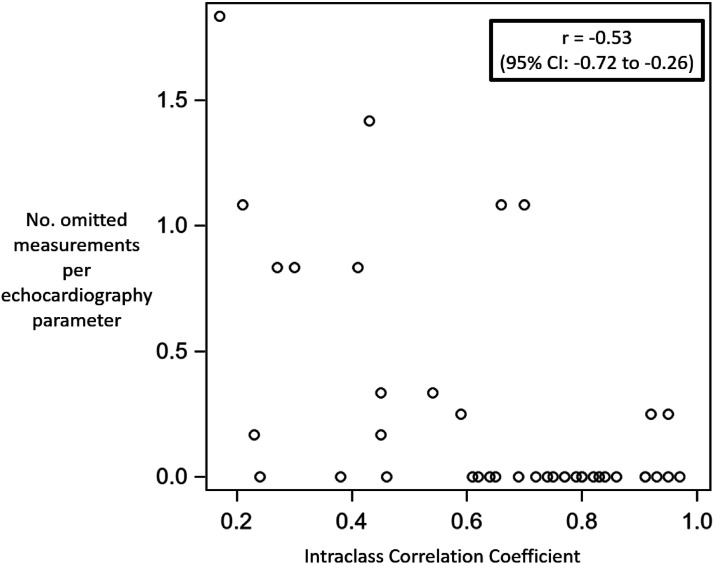
Spearman correlation of the intraclass correlation coefficient and number of measurements omitted per echocardiography parameter.

Among the 84 echocardiogram reviews and 33 possible continuous echocardiography variables to be analyzed per echocardiogram (representing 2772 total potential measurements), the participating neonatologists opted to omit 99 measurements (3.6%). Of these, 21 were appropriately omitted due to non-applicability of the three PDA Doppler-derived gradient measurements in one TNE showing a bidirectional shunt, omitted by all 7 observers. The remaining 78 (2.8%) measurements were omitted due to perceived suboptimal image quality. The median number of omitted measurements per TNE parameter per patient was 0 [IQR 0 – 0.25, range 0–1.1]. There was a moderate negative correlation between the number of omitted TNE measurements per parameter per patient and the ICC for that TNE parameter (Spearman correlation r = −0.53, 95% CI: −0.72 to −0.26, *p* < 0.001) (Figure 2).

## Discussion

In this prospective study of the interobserver variability of TNE measurements of ELGANs with PDA, we identified that among neonatologists with TNE expertise, reliability was excellent for indices of PDA size and shunt volume, left heart dimensions and output, and diastolic flow abnormalities in systemic arteries. These findings support the validity of incorporating these indices in a prospective, multicenter research study. However, the reliability of some indices of LV systolic and diastolic function was variable, with some parameters having poor or moderate reliability when assessed using ICC, though with good reproducibility when assessed using the CV.

To the best of our knowledge, this is the first study to investigate the reliability of a comprehensive set of echocardiography variables for PDA among extremely preterm neonates, evaluated by a large set of expert raters across a national network. Prior reliability studies in preterm neonates with PDA have reported good to excellent reliability in estimates of ductal diameter, left heart dimensions, ventricular outputs, and Doppler flow characteristics of cerebral and splanchnic arteries,^5,16^ though these were single-center studies that included a small number of observers (≤3) and typically investigated only a few parameters (≤6). Comprehensive echocardiography evaluation of PDA has been proposed to improve diagnostic validity and avoid overreliance on a single, or small set of, echocardiography parameters (such as ductal diameter) for clinical decision making.^16,22,23^ While a small number of multicenter studies of limited size have attempted to define a hemodynamically significant PDA by including multiple echocardiography parameters in predictive models, the interobserver reliability of these included parameters has not been previously evaluated.^24^

We identified that several indices of ventricular function had suboptimal reliability, when assessed using the ICC, suggesting caution in incorporating these parameters in predictive analyses. Pulmonary vein velocities on PW Doppler, tissue Doppler imaging (TDI) parameters of LV diastolic performance, and RV indices of ventricular-arterial coupling had poor or moderate reliability. In contrast, prior studies of the reliability of some of these indices in neonates with and without PDA had demonstrated excellent reliability.^25^ There are several potential explanations for this discrepancy. First, our study replicated real-life conditions of providing complete echocardiograms and permitting observer-driven selection of images for analysis, rather than directed analysis of a pre-specified image. Second, our study included a large number of observers practicing across geographically distant settings, which may be more likely to engender measurement variability as compared to few observers working closely in the same site. Third, disease-specific characteristics may have contributed to reduced reliability. For example, tachycardia associated with a large PDA shunt may have resulted in shorter diastolic times and blending of the early (e’) and atrial (a’) velocities on TDI. In addition, antegrade diastolic flow in the main pulmonary artery due to the PDA shunt often obscures the borders of the PW Doppler envelope, decreasing the accuracy of estimates of pulmonary artery acceleration and RV ejection times. Finally, reduced image quality and measurability may have also been related to intensive care supports, such as the provision of high-frequency ventilation. Importantly, there is uncertainty regarding how to adjudicate a minimum reliability threshold for inclusion of borderline parameters in predictive analyses, and this remains an important area for future methodological studies.

In addition, the moderate inverse correlation of the ICC and the number of omitted measurements per echocardiography parameter potentially implies that among echocardiography parameters where some observers chose to omit measurement, the reliability of the parameter measurements, when performed by other observers (who elected not to omit), was reduced. This association suggests that the low reliability of some parameters in our study may have been due, in part, to suboptimal image quality or measurability. Methods of mitigating the impact of omitted measurements in future studies may include defining minimum image quality criteria for inclusion, excluding echo parameters which have a significant frequency of omitted data, and/or use of artificial intelligence^26^ to automate the assessment of image quality to identify low-quality images for exclusion.

We identified discordance in our study among the ICC and CV for some echocardiography indices of LV dimensions and function, which may be related to differences in how these measures are estimated. The variable and inferior ICC for many of the LV functional parameters may in part be explained by the narrow data range in our study population on account of an absence of patients with compromised LV function, which may have exaggerated the influence of operator-related variability. This is supported by the observed low and excellent CV between operators for the same parameters. The ICC estimates how distinguishable subjects are and is calculated as the ratio of the between-subject variance to the total variance. Thus, both larger between-subject heterogeneity and lower random error in measurement may contribute to a higher ICC. In contrast, the within-subject CV, calculated as the ratio of the standard deviation to the mean, determines the closeness of the repeated observations, and is not influenced by between-subject heterogeneity. As a result, echocardiography indices with low between-subject heterogeneity but highly reproducible measurements may have both a low ICC and low CV.

The influence of population heterogeneity on ICC suggests that if studies report only ICC, readers may only make use of the estimate if the population in which the reader intends to use the measurement has similar heterogeneity. While CV has been endorsed as a measure of reproducibility and discouraged as a primary measure of reliability,^27,28^ reporting of both CV and ICC has been suggested to permit readers to judge reliability in different populations,^29^ In cases of measurements for which the overall variability in the study population is low, such as LV systolic function among preterm neonates with PDA, the ICC may falsely show poor reproducibility, and a low CV may be sufficient to infer good reliability.^25^ This also highlights the potential importance of jointly considering the ICC, CV, and heterogeneity of measurements when interpreting measures of reliability and reproducibility in this population. Altogether, our data suggests that although LV function parameters may be valid for multicenter studies of extremely preterm neonates with PDA, there is a scope of further standardization and QA work targeting improvement in the ICC between operators.

In our study, there was excellent agreement in the determination of the presence or absence of holodiastolic flow reversal in the abdominal aorta, and complete agreement in the categorical classification of diastolic flow abnormalities in the celiac artery and MCA, especially when considered as a binary variable of normal (antegrade) or abnormal (reverse or absent). Abnormal diastolic flow in the MCA has been associated with the composite outcome of death or bronchopulmonary dysplasia,^30^ a validation against key outcomes which, when combined with the agreement identified in our study, suggests potential value of incorporating this parameter in clinical practice. In contrast, agreement was reduced for a ternary classification of celiac artery diastolic flow (antegrade, absent, or reverse). Unlike the MCA, the celiac artery moves with diaphragmatic excursion during respiration. The celiac artery Doppler envelope may inadvertently capture adjacent venous flow or incompletely capture the direction of flow during diastole, making it difficult to differentiate between absent and reverse diastolic flow. Further research evaluating the clinical value of a ternary versus binary classification of diastolic flow in systemic arteries is needed to determine if the ternary classification may provide significantly improved discrimination of outcomes to offset the associated reduction in reliability.

Strengths of our study include the use of consecutive clinical echocardiograms to avoid selection bias, the evaluation of a comprehensive set of echocardiography parameters, the wide range in sizes of PDAs evaluated, and the ability of neonatologists to freely navigate the echocardiogram. This pragmatic approach may increase the generalizability of the study by providing a case-mix and analytical approach that broadly reflects contemporary clinical practice.

Our study has several limitations. First, although tele-echocardiography and remote analysis are increasingly being used in clinical practice,^31,32^ its use as a research tool has not been previously validated. While the videoconferencing interface increased the feasibility of this national pragmatic study, an impact on echocardiography measurements cannot be excluded. Second, intraobserver and test–retest reliabilities were not evaluated as these were identified as potentially having a minimal impact on the validity of multicenter studies pooling echocardiography data as covariates in predictive models of clinical outcomes. Third, the reliability of categorical classifications of ductal size (e.g., “moderate” vs “large”) was not evaluated; we elected to focus predominantly on continuous parameters that potentially provide greater statistical power for discrimination in predictive models. Finally, the variation that may originate from differences in quality during image acquisition in different centers or by different sonographers was not assessed or accounted for in this study, both of which may be sources of bias. Our results may only be valid in the context of a highly standardized imaging protocol and measurement techniques, as was achieved across our national group prior to undertaking this study.

Although our study evaluated the interobserver reliability of manually estimated echocardiography parameters of patent ductus arteriosus, artificial intelligence (AI) and automated echocardiography quantification methods hold promise as efficient and reliable approaches that may address some of the limitations identified. Test–retest reproducibility of AI measurements has been reported to be superior to manual measurements in inter-observer scenarios and non-inferior in intra-observer scenarios involving adults, while taking less time to complete.^33^ Similarly, automated pediatric left ventricle analysis has recently been demonstrated to be feasible, with machine learning-enabled image analysis reducing analysis time and producing results comparable to traditional methods.^34^ Perhaps most innovatively, novel neural network models with adaptive ranking and structure-aware learning now enable automated methods of assessing image quality by deciphering the intricate relationships between an image’s semantic structure and quality.^26^ Although integration of AI in neonatal echocardiography analysis is in its infancy, such methods may help overcome the limitations of reduced image quality or reliability among some parameters.

## Conclusion

Interobserver reliability for TNE evaluation of ELGANs with PDA was excellent for PDA size and gradient, and good-to-excellent for most indices of LV dimension and output, and diastolic flow abnormalities. These findings support the validity of incorporating these TNE indices in prospective, multicenter research.

## ORCID iDs

Dany E Weisz https://orcid.org/0000-0002-7335-8674

Laura Thomas https://orcid.org/0000-0002-6401-209X

Gabriel Altit https://orcid.org/0000-0001-5141-0964

Nadya Ben Fadel https://orcid.org/0000-0002-7938-2499

Deepak Louis https://orcid.org/0000-0002-8535-4208

Joseph Y Ting https://orcid.org/0000-0002-5246-8823

Aimann Surak https://orcid.org/0000-0003-3477-3741

Poorva Deshpande https://orcid.org/0000-0001-7228-0118

Bonny Jasani https://orcid.org/0000-0001-6741-0782

Sagee Nissimov https://orcid.org/0000-0001-7873-5224

Faith Zhu https://orcid.org/0000-0002-2263-4448

Prakesh Shah https://orcid.org/0000-0002-9920-0488

Amish Jain https://orcid.org/0000-0002-1413-2966

## Appendix

### Abbreviations

AI: artificial intelligence
ELGANs: extremely low gestational age neonates
GA: gestational age
LV: left ventricle
LVO: LV output
NICU: neonatal intensive care unit
PDA: patent ductus arteriosus
RV: right ventricle
TNE: targeted neonatal echocardiography.
